# The Prince of Wales's Hospital Fund for London

**Published:** 1897-07-17

**Authors:** 


### THE PRINCE OF WALES'S HOSPITAL FUND FOR LONDON.

### VISIT OF T.R.H. THE DUKE AND DUCHESS OF YORK TO THE BANK OF ENGLAND.

On Friday, July 9th, the Duke and Duchess of York visited the Bank of England to witness the destruction of the plates from which the stamps issued for the benefit of the Prince of Wales's Hospital Fund were printed. It will no doubt be remembered that as soon as the printing of the stamps was completed the plates were consigned to the strong rooms of the Bank, under the official seals of the Governor and chief cashier, which were affixed in the presence of several of the honorary officials of the Fund, until a convenient opportunity presented itself for their destruction. At this function H.R.H. the Duke of York, who, as President of the London Philatelic Society, takes a great interest in the matter, graciously signified his willingness to be present.

Their Royal Highnesses, who were attended by Sir Charles Cust and Lady Mary Lygon, on arrival at the Lothbury entrance, were received by the Governor, Mr. Hugh Colin Smith; the Deputy-Governor, Mr. S. Steuart Gladstone; the Chief Cashier, Mr. H. G. Bowen, and the Acting Secretary, Mr. Kenneth Grahame, of the Bank of England, and also by the following members of the Organising Committee of the Prince of Wales's Hospital Fund: Lord Rothschild, Lord Rowton, Sir Henry Burdett, K.C.B., the Right Hon. C. Stuart Wortley, Q.C., M P., Sir Savile Crossley, and Mr. J. G. Craggs.

There were also present: Mr. J. S. Purcell, C.B., Controller of Stamps at Somerset House; Mr. Thomas De la Rue, of Messrs. De la Rue and Co., who carried out the printing of the stamps, and who so kindly prepared the design, engraved the plates, and presented them to the Fund free of charge; Mr. G. H. Miles and Mr. Joseph Shaylor, of Messrs. Simpkin Marshall and Co., the wholesale distributors of the stamps, whose task in dealing with the issue has been anything but a light one; and Mr. S. G. Wilkinson, of the Oxford Bible Warehouse, who have used a very large number of the stamps in their Commemoration Bibles and Prayer-Books; and representatives of the various philatelic societies.

In the die sinking department, where the plates were deposited, and which is under the charge of Mr. F. C. Smith (who has been in the service of the Bank over forty years), all the dies and water-marking plates are designed and made for the Bank-notes, Postal Orders, Indian notes, Exchequer bills, and any other papers requiring a water-mark, and Mr. Smith takes a pride in showing a paper mould (from which the water-mark is impressed upon the paper whilst in course of manufacture) of a fine Damascus pattern, the process for making which was patented by his father, Mr. John Smith, in 1849, the mould being shown at the Great Exhibition of 1851.

At the benches in this room were stationed in readiness three expert workmen, each provided with an enormous two-inch file or rubber weighing sixteen pounds, and the steel plates were fixed in position on the benches and the workmen set to work with a will to obliterate every line of the delicate tracery constituting the design of the plates. The destruction of the steel plates being complete, the attention of the workmen was then directed to the original matrix and die, and in a short space of time the designs were ground level with the surfaces of the matrix and die. The defaced plates and designs were once again consigned to the charge of the Bank officials.

Their Royal Highnesses then proceeded to the Governor's room, where the official certificates of the destruction of the plates was signed by their Royal Highnesses the Duke and Duchess of York, Mr. H. C. Smith, the Governor of the Bank of England, Mr. J. S. Purcell, C.B, Controller of Stamps, and the following members of the Organising Committee of the Prince of Wales's Hospital Fund: Lord Rothschild, Lord Rowton, Sir Henry Burdett, K.C.B., the Right Hon. C. Stuart Wortley, Q.C., M.P., Sir Savile Crossley, and Mr. J. G. Craggs. The certificate is as follows:—

"Bank of England.

"Certificate of the destruction of the dies and plates employed in the production of the Prince of Wales's Hospital Fund Stamps.

"We, the undersigned, hereby certify that the whole of the dies and plates used in the production of these stamps were this day destroyed in our presence."

(Here follow the signatures as above.)

The Governor of the Bank then said: On behalf of the committee of the Prince of Wales's Fund I beg to thank your Royal Highness and the Duchess for coming here to-day to see the plates destroyed. Perhaps you will kindly certify to those present that you are quite satisfied that no more stamps can be printed from the plates.

The Duke of York replied: It has interested both the Duchess and myself a great deal to come to-day and see the destruction of the dies and plates of the stamps which are issued in connection with the Prince of Wales's Hospital Fund, and I trust that the remainder of the stamps will be soon sold as I think there can be no more appropriate souvenir of Her Majesty's Diamond Jubilee than these stamps. His Royal Highness added that the defaced plates and dies might form an interesting exhibit at the forthcoming Philatelic Exhibition which he is to open in London on the 22nd inst.

